# Mothers/caregivers' knowledge of routine childhood immunization and vaccination status in children aged, 12-23 months in Ilorin, Nigeria

**DOI:** 10.4314/ahs.v23i4.61

**Published:** 2023-12

**Authors:** Solomon O Ariyibi, Ayodele I Ojuawo, Rasheedat M Ibraheem, Folake M Afolayan, Olayinka R Ibrahim

**Affiliations:** 1 Department of Paediatrics, University of Ilorin Teaching Hospital, Ilorin, Kwara State, Nigeria; 2 General Hospital, Kwara State Hospital Services, Ilorin, Nigeria

**Keywords:** Vaccination uptake, routine childhood immunization, Mothers' knowledge

## Abstract

**Background:**

Immunization has averted millions of hospitalizations and deaths from vaccine-preventable diseases. It is a strong public health tool for childhood infection control and prevention. Many mothers are aware of routine immunization but with doubtable knowledge.

**Objectives:**

This study determined the mothers/caregivers' knowledge of routine childhood immunization and vaccination status of their children, aged 12-23 months in Ilorin East Area of Kwara State, Nigeria. It also identified some of the socio-demographic factors associated with good knowledge status of the mothers/caregivers.

**Methods:**

This was a community-based cross-sectional study, carried out between December, 2019 and January, 2020, among 456 mothers / caregivers-children's pairs. Subjects were recruited using multistage cluster sampling technique. Data were collected using a pretested, semi-structured, interviewer-administered questionnaire.

**Results:**

Up to 98.0% of the respondents were aware of childhood immunization with healthcare providers (92.1%) being their major source of information. Majority of the respondents (85.3%) had good knowledge of immunization, defined by a score ≤6 out of the 10 questions tested. There was a significant relationship between respondents' knowledge and full vaccination status of the children (p=0.001). The significant factors associated with good knowledge from binary logistic regression were mothers / caregivers' age >30 years, antenatal clinic attendance and at least secondary education (OR, p value = 10.60, 0.013; 8.50, <0.001; and 3.98, <0.001 respectively).

**Conclusion:**

Mothers / caregivers' knowledge on immunization was good and this positively affected the full vaccination status of their children. There is a need to sustain female education and encourage antenatal clinic attendance, as tools to improve childhood immunization.

## Introduction

Immunization remains one of the most economical public health interventions that has significantly reduced the burden of childhood infectious diseases all over the world. It has prevented millions of hospitalizations and deaths from vaccine-preventable diseases for over three decades.^1^,^2^ Despite this huge benefit, many children especially in the developing countries like Nigeria are not fully immunized, making them susceptible to vaccine-preventable deaths.

The expanded Programme on Immunization (EPI) was launched by the World Health Organization (WHO) in 1974. However, in order to promote its effectiveness in Nigeria, it was restructured and renamed National Programme on Immunization (NPI) in 1996. According to the current schedule, a child in Nigeria should receive BCG, OPV_0_ and HBV at birth; Penta_1_ (DPT, HBV and Hib), OPV_1_, PCV_1_ and Rota_1_ at 6 weeks; Penta_2_, OPV_2_, PCV_2_ and Rota_2_ at 10 weeks; Penta_3_, OPV_3_, PCV_3_ and IPV at 14 weeks; Measles-1, Yellow fever and meningitis A at 9 months; and Measles-2 at 15 months.

Uptake of these vaccines by children depend on many factors, including maternal knowledge and attitude, socio-cultural characteristics of the family as well as health facility-related factors. Studies have shown that mothers' knowledge of routine immunization significantly affects their uptake of immunization services.^3^,^4^

In Nigeria, Northern states, including Kwara, the site of the present study, have low vaccination uptake compared with other regions of the country.^5^ The Nigerian Demographic and Health Survey (NDHS) 2018 revealed that only 26% of children in Kwara State had received all recommended vaccines, which is far below the global target of 90%.^6^ Many mothers are aware of routine immunization programme, especially in the semi-urban areas, but with doubtable knowledge. Therefore, this study was conducted to determine the maternal knowledge of routine immunization and its determining factors, as well as the vaccination status of children aged 12-23 months in Ilorin, North-central Nigeria.

## Materials and methods

### Study design

We conducted a community-based cross-sectional study that involved children aged 12-23 months and their mothers/caregivers.

### Study setting

This study was carried out in Ilorin East Local Government Area of Kwara State, North-central Nigeria from December, 2019 to January, 2020. According to the 2006 census, Ilorin East has a population of 207,462 people and children aged 12-23 months constituted about 3.5% of the total population.^7^ The local government has 12 wards, 16 primary health centres, and one comprehensive health centre.

### Sample size determination and sampling technique

A total of 456 children aged 12-23 months was calculated using the formula for qualitative variable with 95% confidence interval, 5% margin of error and 58% estimated coverage rate according to a study in Southwestern Nigeria.^8^

Eligible children were sampled using a multistage cluster sampling technique. In stage one, simple random sampling was done to select four wards out of the 12 wards in Ilorin East Local Government Area. In stage two, four communities were selected from each of the four selected wards by balloting, making a total of 16 communities. A proportionate allocation was used to allocate the sample size to each of the selected communities using the estimated size of the target population, i.e., number of children aged 12-23 months (3.5% of the total population of that community as stated in the year 2019 Kwara State harmonized microplan for immunization, Ilorin East Local Government Area). The population of each of the communities was also obtained from the microplan document. The sample size in each community was allocated as follows:

[Number of children aged 12-23months in each community /total number of children aged 12-23 months in the 16 communities] multipliplird by N.

Where N is the calculated sample size, 456.

In stage three, the first house to start the study from in each community was randomly selected and subsequent houses were selected contiguously in the right direction. All eligible children in all the households in the houses visited were recruited until the sample size was completed. One child was recruited from each mother / caregiver. Mothers / caregivers of the recruited children were interviewed. The four wards where children were recruited from are Oke-Ose/Oke Oyi (167 children), Zango (137 children), Gambari (92 children) and Maya (60 children). The inclusion criteria were children aged 12-23 months whose mothers/caregivers consented to the study, while children < 12 or > 23 months were excluded.

### Data collection

A pretested semi-structured interviewer-administered questionnaire was used for obtaining data. The research instrument included sections on socio-demographic characteristics of mothers/caregivers and their children, knowledge of mothers / caregivers on routine childhood immunization and vaccine-preventable diseases, as well as vaccination uptake for each antigen by card or certificate. Maternal/caregiver's knowledge of routine immunization was assessed based on responses to ten (10) questions in the research instrument. Each correctly answered question was awarded a score of 1, while a wrong or unknown answer was awarded 0 (scores were indicated after each question), with a total score of 10. The correct answer was assessed by the researcher or a trained research assistant and scores were assigned as appropriate. Mothers / caregivers who scored < 6 were adjudged to have poor knowledge of RI while mothers / caregivers with ≥ 6 were adjudged to have a good knowledge of routine childhood immunization. This is similar to the method used in determining vaccination coverage in rural Nigeria.^9^

The immunization cards or certificates were checked to confirm information on vaccination provided by the mothers / caregivers. Information on other aspects of the study instrument was also obtained from the mothers / caregivers. The vaccination status of the children following uptake was grouped into three, namely: fully immunized, partially immunized and not immunized. A fully immunized child is any child who had received one dose each of BCG and HBV0, four doses of OPV, three doses each of pentavalent and pneumococcal conjugate, and one dose each of IPV, measles and yellow fever vaccine by 12 months of age; partially immunized child is any child who missed any one of the above doses; and an un-immunized child is any child who had not received any vaccine by 12 months of age.^5^

### Data analysis

Data were entered into a computer, sorted and analysed using a Statistical Package for Social Sciences (SPSS) version 23. Descriptive statistical analysis was done and the results were summarized as frequencies and proportions for categorical variables, and mean and standard deviation (SD) for continuous variables. Chi square was used for bivariate analysis to assess the association between categorical variables. Then, all variables that showed statistical significance in the bivariate analysis for mothers / caregivers' socio-demographic characteristics were included in the binary logistic regression to determine the factors that were associated with mothers' good knowledge status. A p-value < 0.05 was considered statistically significant.

## Results

### Characteristics of the mother/caregiver-children's pairs

A total of 456 mothers / caregivers of children aged 12-23 months old were interviewed. The mean (SD) age of the mothers / caregivers was 30.9 (5.3) years, while that of the children was 17.8 (3.2) months. The male to female ratio was 1.2:1. Caregivers constituted two percent of the respondents. Majority of the respondents (84.9%) had some form of formal education (primary, secondary or tertiary) Table I.

**Table I:** Characteristics of the mother/caregiver-children's pairs

Variables	Frequency	Percent
**Sex of children**		
Male	250	54.8
Female	206	45.2
**Child's birth order**		
1^st^	63	13.8
2^nd^-4^th^	374	82.0
≥ 5^th^	19	4.2
**Child age group (months)**		
12 – 15	123	27.0
16 – 19	196	43.0
20 – 23	137	30.0
**Hospital birth**		
Yes	362	79.4
No	94	20.6
**Age group of Mother / Caregiver (years)**		
≤20	7	1.5
21 – 30	233	51.1
31 – 40	198	43.4
41 – 50	18	3.9
**Marital Status**		
Single	16	3.5
Married	440	96.5
**Education**		
No formal education	69	15.1
Primary education	102	22.4
Secondary education	200	43.9
Tertiary education	85	18.6
**Employment status**		
Employed	100	21.9
Unemployed	356	78.1
**Relationship to child**		
Mother	447	98.0
Caregiver	9	2.0
**ANC attendance**		
Yes	433	95.0
No	23	5.0

**ANC: Antenatal care.**

### Mothers / caregivers' awareness of routine childhood immunization

**Figure 1:**
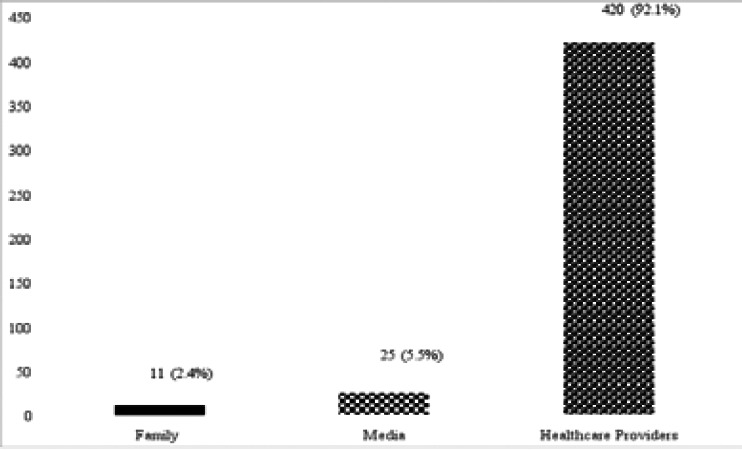
Sources of mothers / caregivers' knowledge of childhood immunization

Four hundred and forty-seven (98.0%) of the women interviewed were aware of childhood routine immunization. Four hundred and twenty (92.1%) stated that their source of knowledge was from health care providers at the antenatal care clinics and other health service points (Figure 1).

### Mothers / caregivers' knowledge of routine childhood immunization

Highest proportion of correct response on mothers/caregivers' knowledge of childhood routine immunization (RI) was reported for knowledge of the purpose of immunization at 97.1% while the lowest proportions were obtained for age at which child was to receive the third dose of routine immunization and the number of times oral polio vaccine was to be taken, both at 46.1% (Table II). Overall, 389 (85.3%) respondents had good knowledge of routine immunization while 67 (14.7%) had poor knowledge.

**Table II:** Maternal/Caregiver knowledge of childhood routine immunization (RI)

Questions	N	Correct (%)	Incorrect (%)
1. What is the purpose of immunization?	456	443(97.1)	13(2.9)
2. Are all childhood diseases (including malaria and HIV) vaccine-preventable?	456	236(51.8)	220(48.2)
3. When should vaccination begin for a child?	456	421(92.3)	35(7.7)
4. At what age should a child receive the 3rd dose of routine immunization?	456	210(46.1)	246(53.9)
5. At what age should a child receive the last dose of routine immunization?	456	379(83.1)	77(16.9)
6. How many times should OPV be taken in RI	456	210(46.1)	246(53.9)
7. What is the total number of visits to the health facility for a child to obtain all vaccines?	456	370(81.1)	86(18.9)
8. Mention at least 3 vaccine-preventable diseases	456	331(72.6)	125(27.4)
9. Mention at least 3 symptoms of vaccine-preventable diseases	456	302(66.2)	154(33.8)
10. Mention at least 2 adverse effects of vaccination	456	404(88.6)	52(11.4)

**NB:** Each correct answer attracted 1 mark, while incorrect answers attracted 0.

### Mothers / caregivers' socio-demographic characteristics and their RI knowledge status

Table III shows that mothers / caregivers' age, educational status, employment status, and antenatal clinic attendance had significant association with good knowledge of RI (p values 0.011, <0.001, 0.001 and <0.001 respectively). In the binary logistic regression analysis (Table IV), antenatal clinic attendance, higher level of education (at least secondary) and mothers / caregivers' age above 30 years were significantly associated with a good knowledge of routine immunization.

**Table III:** Mothers / caregivers' socio-demographic characteristics and their RI knowledge status

Variable	Knowledge status		Total	*χ* ^2^	p value
	Good	Poor			
**Relationship to child**					
Mother	381 (85.2)	66 (14.8)	447	0.03^Y^	0.865
Caregivers	8 (88.9)	1 (11.1)	9		
**Age (years)**					
≤20	3 (42.9)	4 (57.1)	**7**	11.21^Y^	**0.011** *
21-30	192 (82.4)	41 (17.6)	233		
31-40	177 (89.4)	21 (10.6)	198		
41-50	17 (94.4)	1 (5.6)	18		
**Religion**					
Islam	345 (85.0)	61 (15.0)	406	0.33	0.569
Christianity	44 (88.0)	6 (12.0)	50		
**Marital status**					
Single	3 (18.8)	13 (81.3)	16	0.01^Y^	0.916
Married	64 (14.5)	376 (85.5)	440		
**Education**					
No formal education	47 (68.1)	22 (31.9)	69	41.36	**<0.001** *
Primary education	76 (74.5)	26 (25.5)	102		
Secondary education	184 (92.0)	16 (8.0)	200		
Tertiary education	82 (96.5)	3 (3.5)	85		
**Employment status**					
Employed	96 (96.0)	4 (4.0)	100	11.69	**0.001** *
Unemployed	293 (82.3)	63 (17.7)	356		
**Antenatal care attendance**					
Yes	381 (88.0)	52 (12.0)	433	45.18^Y^	**<0.001** *
No	8 (34.8)	15 (65.2)	23		

***χ*** ^**2**^ **: Chi square test; Y: Yates corrected Chi square**

* ***p*** **value < 0.05**

**Table IV:** Multivariate logistic regression of some socio-demographic characteristics of mothers / caregivers and RI knowledge status

Parameters	Good knowledge OR (95% CI)	p value
**ANC attendance**		
Yes versus No	8.50 (3.16-22.89)	**< 0.001** *
**Employment status**		
Employed versus Unemployed	3.06 (0.99-9.40)	0.051
**Mothers' level of education**		
≥Secondary versus	3.98 (2.14-7.42)	**<0.001** *
<Secondary and none	1	
**Mothers' age**		
>30 years	10.63 (1.66-68.19)	**0.013** *
21-30 years	5.29 (0.85-32.81)	0.073
<20 years^REF^	1	

* *p* value < 0.05

OR: Odd ratio, CI: Confidence interval, ANC: Antenatal care.

### Vaccination uptake prevalence of the various antigens and children's vaccination status

**Figure 2:**
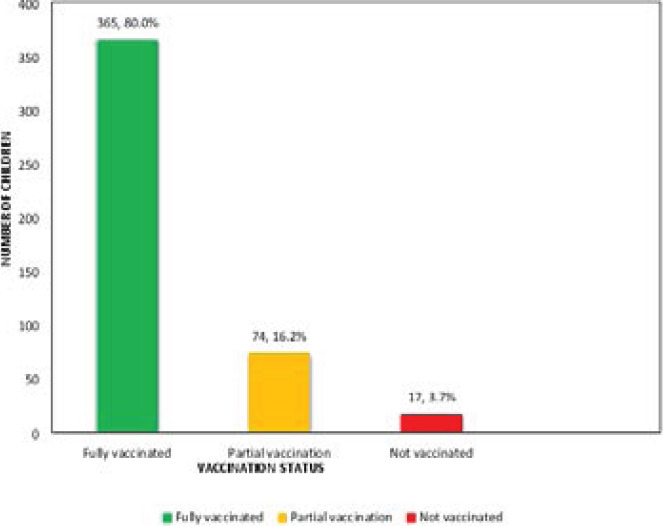
Vaccination status of children studied

Of the 456 children that were recruited into the study, 439 children (96.3%) had immunization cards while 17 children (3.7%) had no immunization cards. By verification of antigens from cards, 365 children (80.0%) received all vaccines. The highest uptake prevalence of 100% was identified for BCG, OPV0, HBV0, OPV1 and Penta1 while yellow fever vaccine had the lowest uptake prevalence of 83.1%. Figure 2 shows the vaccination stats of the children.

### Mothers / caregivers' knowledge of routine immunization and vaccination status of their children

Of the 439 children that were vaccinated with one or more antigens, 387 (88.2%) of their mothers/caregivers had good knowledge of immunization (p < 0.001). Fifteen out of the 17 unimmunized children (88.2%) belong to mothers/caregivers with poor knowledge of immunization (Table VI). Comparing mothers / caregivers' knowledge status with the levels of vaccination, the mothers of 330 children (90.4%) with full vaccination had a significantly higher knowledge of childhood immunization compared with 57 mothers (77.0%) of children with partial vaccination status (p value = 0.001).

## Discussion

Childhood immunization remains a veritable tool towards combating the childhood vaccine preventable diseases. This study shows that the general awareness of childhood immunization from various sources was good (98%) with health care providers in and outside antenatal care clinics being their major source (92.1%). Also, 95.0% of the respondents attended antenatal care clinics which is significantly associated with a good knowledge of routine immunization in the present study as it provides mothers with access to information on childhood immunization by the health care workers. The finding in this study is similar to the level of awareness reported from Oyo State, South-Western Nigeria (98.1%), Edo State, South-Southern Nigeria (99.9%), a hospital facility based study in Urban Lagos Nigeria (93.8%) and 96.0% reported in Ethiopia; health care workers being their major source of knowledge^8^-^11^ The high level of awareness observed in this study may be attributed to the fact that information on childhood immunization is often discussed and reiterated at visits to the antenatal care clinics. Also, opportunity at various clinics and outpatients may also provide information on the benefit of routine childhood immunization viz-a-viz protection against certain infectious diseases.

Assessment of respondents' knowledge of childhood immunization revealed that majority (85.3%) had good knowledge and which may account for the reason a large percentage of the children were fully vaccinated in this study. It is also worthy of note that close to half of the respondents (48.2%) felt all childhood diseases are vaccine-preventable. This may be due to the fact that most health care providers do not specifically clarify that not all childhood diseases are vaccine-preventable during health talks, especially at the antenatal care clinics. Hence, most mothers assumed that it protects a child against all diseases, including HIV and malaria, as observed in this study. The study findings on the maternal knowledge of childhood immunization are similar to that in a study in South-Western Nigeria^8^ where 41.3% of their respondents erroneously believed that childhood immunization prevents HIV and 5.2% held the fact that local herbs are good substitute for immunization. This study also shows that 46.1% of the respondents did not know the age-timing of the vaccines and number of times to obtain the multiple dose vaccines (e.g., OPV) for their children. The mothers/caregivers only take their children to the clinics according to appointments without knowing which vaccines were being administered. This observation calls for the need to educate and enlighten mothers/caregivers on the vaccines and their purposes during visits to the immunization clinics. This will further promote vaccine acceptance and prevent erroneous omission or missed opportunity at the health facility.

Mothers / caregivers' age, educational status, employment status, and antenatal clinic attendance showed significant association with their knowledge on childhood routine immunization in the present study, similar to several other studies.^3^,^12^-^16^. However, mothers being employed is not a significant predictor of good knowledge of immunization. Mothers / caregivers with at least secondary education have almost 4-fold likelihood of having good knowledge of immunization when compared with those with lesser or no formal education as shown in the current study. This finding is in consonance with several other findings where it was reported that mothers with higher level of education had better knowledge of immunization.^4^,^15^-^17^ This is because being educated and even a high level of education improves awareness and understanding of the efficacy of childhood immunization.

Other factors like relationship of respondents to child, their religion, and marital status did not significantly influence their knowledge on immunization, similar to other studies.^3^,^4^,^10^

Maternal knowledge of childhood immunization showed a significant association with vaccination uptake in this study. A large proportion of children with full vaccination (90.4%) were from mothers with good knowledge of immunization. Several other studies have also shown that mothers' knowledge of the relevance and schedule of vaccination has a strong relationship with complete vaccination status of children.^11^,^13^,^14^,^18^ Most of the unimmunized children in this study (88.2%) were from mothers with poor knowledge of childhood immunization. The high vaccination uptake and full vaccination status in this study can therefore be linked to the respondents' good knowledge of immunization.

## Conclusion

The level of awareness of childhood immunization among respondents in the study was high and the respondents' knowledge of childhood routine immunization was good. Old mothers / caregivers, antenatal care clinic attendance, high level of education and being employed were the significant factors identified to be associated with good knowledge of immunization.

## Limitation of this study

There were few instances where houses dominated by Hausa or Fulani who could not speak English or Yoruba were skipped due to absence of an interpreter. This may create some bias in this study. However, they were very few and may not have affected the results of this study significantly. Also, the questionnaire was designed in English Language with no back translation.

## Figures and Tables

**Table V:** Maternal knowledge of childhood immunization and vaccination status

	Vaccination status				

	Vaccinated (full & partial)	Not vaccinated	Total	*χ* ^2^	p value
	n =439 (%)	n =17 (%)	N=456		
**Maternal Knowledge of** **childhood immunization**					
Good	387 (88.2)	2 (11.8)	389	70.22	**<0.001** *
Poor	52 (11.8)	15 (88.2)	67		
	**Vaccinated (n = 439)**				
	**Full, n = 365 (%)**	**Partial, n = 74 (%)**			
Good	330 (90.4)	57 (77.0)	387	10.56	**0.001** *
Poor	35 (9.6)	17 (23.0)	52		

* ***p* value < 0.05**

***χ*** ^**2**^ **: Chi square.**
